# Critical Link between Calcium Regional Heterogeneity and Atrial Fibrillation Susceptibility in Sheep Left Atria

**DOI:** 10.3390/jcm12030746

**Published:** 2023-01-17

**Authors:** Barbara C. Niort, Alice Recalde, Caroline Cros, Fabien Brette

**Affiliations:** 1 Centre de Recherche Cardio-Thoracique de Bordeaux (CRCTB), Inserm U1045, Univeristé de Bordeaux, F-33000 Bordeaux, France; 2 IHU Liryc, Electrophysiology and Heart Modeling Institute, F-33600 Pessac, France; 3 Phymedexp Inserm, CNRS, Université de Montpellier, CHRU, F-34295 Montpellier, France

**Keywords:** atrial fibrillation, animal model, pulmonary vein, calcium, optical mapping

## Abstract

Background: Atrial fibrillation is the most sustained form of arrhythmia in the human population that leads to important electrophysiological and structural cardiac remodeling as it progresses into a chronic form. Calcium is an established key player of cellular electrophysiology in the heart, yet to date, there is no information that maps calcium signaling across the left atrium. Objective: The aim of this study is to determine whether calcium signaling is homogenous throughout the different regions of the left atrium. This work tests the hypothesis that differences across the healthy left atrium contribute to a unique, region-dependent calcium cycling and participates in the pro-arrhythmic activity during atrial fibrillation. Methods: An animal model relevant to human cardiac function (the sheep) was used to characterize both the electrical activity and the calcium signaling of three distinct left atrium regions (appendage, free wall and pulmonary veins) in control conditions and after acetylcholine perfusion (5 μM) to induce acute atrial fibrillation. High-resolution dual calcium-voltage optical mapping on the left atria of sheep was performed to explore the spatiotemporal dynamics of calcium signaling in relation to electrophysiological properties. Results: Action potential duration (at 80% repolarization) was not significantly different in the three regions of interest for the three pacing sites. In contrast, the time to 50% calcium transient decay was significantly different depending on the region paced and recorded. Acetylcholine perfusion and burst pacing-induced atrial fibrillation when pulmonary veins and appendage regions were paced but not when the free wall region was. Dantrolene (a ryanodine receptor blocker) did not reduce atrial fibrillation susceptibility. Conclusion: These data provide the first evidence of heterogenous calcium signaling across the healthy left atrium. Such basal regional differences may be exacerbated during the progression of atrial fibrillation and thus play a crucial role in focal arrhythmia initiation without ryanodine receptor gating modification.

## 1. Introduction

Cardiac arrhythmias are major causes of morbidity and mortality [1], with atrial fibrillation (AF) being the most prevalent cardiac arrhythmia [2]. AF is classified as paroxysmal (self-limiting), persistent, and longstanding persistent (continuous for >7 days and >1 year, respectively). AF itself induces AF-promoting remodeling, largely through the effects of a very rapid atrial rate, often referred to as ‘AF begets AF’ [3].

AF is initiated by the presence of one or more hyper-excitability sources, usually located within the left atria (LA), that lead to asynchronous and chaotic myocardial contractions. Those ectopic foci can sustain AF as a driver and may trigger reentries (rotors, [4]) in a vulnerable substrate characterized by slow and inhomogeneous conduction and short effective refractory periods. AF can be managed with pharmacotherapy, but current drugs fall short of addressing relevant needs in the prevention and treatment of AF. Ablation therapy (catheter ablation, [5]) is now a well-established therapeutic option for certain groups of patients with AF, especially in paroxysmal cases where reentries occur mainly in the pulmonary veins (PV) region [6]. However, in these patients, there is an increased risk of thromboembolic stroke and heart failure [7]. There is a clear need to improve therapies, which may lead to personalized medicine [8].

Several cellular mechanisms for AF have been described at the electrical level [9]. The electrical activity of the heart is governed by an intricate system of ion channels, transporters, intracellular organelles, and signaling pathways. Indeed, calcium (Ca^2+^) regulation is a major determinant of cellular electrophysiology in the heart [10]. It is now well-established that Ca^2+^ handling abnormalities play numerous roles in cardiac arrhythmogenesis [11]. Triggered activity can result from early- or delayed after-depolarization (EADs and DADs, respectively). EADs occur with prolonged repolarization, allowing the L-type Ca^2+^ channels to recover from voltage/Ca^2+^-dependent inactivation and produce a secondary depolarization before full AP-repolarization. DADs result from spontaneous diastolic sarcoplasmic reticulum Ca^2+^ release events that activate the Na^+^/Ca^2+^ exchanger (NCX), producing a depolarizing transient-inward current. If membrane depolarization is sufficiently large for the cell’s activation to reach the threshold, an action potential (AP) is triggered, which can produce focal ectopic firing. In the atrial myocyte, these ectopic foci can either trigger AF-maintaining re-entry in a vulnerable substrate or, if they fire repeatedly, act as AF-maintaining drivers [12].

These detailed mechanistic investigations have been performed in isolated cardiac myocytes, allowing for precise Ca^2+^ concentration measurement and quantification. However, these single-cell studies provide little insight into the spatially heterogeneous nature of sarcoplasmic reticulum Ca^2+^ homeostasis and how this affects the initiation and/or maintenance of AF. Thus, it is difficult to directly extrapolate findings in isolated cells to atrial tissue. It is even more critical to study these parameters at the tissue level in order to understand AF since specific regions of the atria (e.g., the PV) are more likely than others to be sources of arrhythmogenic activity that cause AF. Albeit there is now evidence that Ca^2+^ mediates various types of cardiac arrhythmias [13], there is a clear need to investigate Ca^2+^ cycling in relation to electrophysiology to provide first hints in the fibrillatory process during AF.

High-resolution optical mapping techniques have provided valuable information on the spatial resolution of the dynamics of cardiac electrophysiology [14], particularly in atrial tissue from animal models of AF [15]. Rotors, reentrant arrhythmias, rotors giving rise to spiral waves, and the role of the epicardium vs. the endocardium have been clearly defined as tissular mechanisms [16,17,18]. In contrast, less is known regarding the spatial and temporal heterogeneities of voltage and [Ca^2+^]_i_ in the atria due to the technical difficulty of simultaneously recording APs and [Ca^2+^]_i_ from multiple sites with sufficient spatial resolution and speed. Several dual-optical mapping experiments have been performed in rodents [19,20]. However, rodent models do not realistically mimic the human electrophysiological phenotype in some important respects (e.g., basal heart rate and ionic currents governing repolarization) and certainly do not come close to human atrial dimensions, crucial for reentry mechanisms. By contrast, large-animal models exhibit heart rhythm, atrial AP morphology, and heart size that are similar to humans. The sheep is, therefore, a translationally relevant model to study AF since it can recapitulate the disease phenotype in humans. However, to the best of our knowledge, dual optical mapping of V_m_ and [Ca^2+^]_i_ was performed only once to study AF in the context of ischemia in sheep [21]. Interestingly, the authors showed that dantrolene, a ryanodine receptor stabilizer, suppressed AF episodes. Whether the same occurs in the context of acute AF is currently unknown.

In this study, we report for the first time the simultaneous mapping of V_m_ and [Ca^2+^]_i_ across the surface of the LA from a translationally relevant ovine animal model, and we use this novel approach to characterize V_m_ and [Ca^2+^]_i_ in three different regions of the LA. This is crucial because, although PV is the main site for ectopic beats in paroxysmal AF, ~30% arise from other areas of the LA, more specifically the free wall (FW) [22] and the left atrial appendage (LAA) [23]. We mimic paroxysmal AF with acetylcholine perfusion and/or electrical bursts. Our findings show that the Ca^2+^ transient decay was significantly different depending on the region paced and recorded. AF was induced when PV and LAA regions were paced but not the FW region. Dantrolene (a ryanodine receptor blocker) did not reduce AF susceptibility.

## 2. Materials and Methods

### 2.1. Tissue Preparation

Young female adult sheep aged 12–18 months weighing 46 ± 4 kg (N = 21) were premedicated with ketamine (20 mg/kg) and acepromazine (0.02 mL/kg) and anesthetized with propofol (2 mg/kg) and maintained under isoflurane, 2%, in air/O2 (50/50%) after intratracheal intubation. Sheep were euthanized by intravenous injection with pentobarbital (30 mL/50 kg), and hearts were rapidly excised, cannulated, and flushed with cold (4 °C) cardioplegic solution containing (in mmol/L) NaCl, 110; CaCl_2_, 1.2; KCl, 16; MgCl_2_, 16; NaHCO_3_, 10; and glucose, 9.01; supplemented with heparin sodium (2.5 UI/mL). The LA was dissected, cannulated by the left circumflex coronary artery, and turned inside out in order to expose the endocardial surface, especially the pulmonary vein muscle sleeves. Perfusion leaks at cut surfaces were carefully tied-off, and preparations were mounted on a homemade frame. LAs were submerged and perfused (20 mL/min) with a warm (38 °C) saline solution containing (in mmol/L): NaCl, 130; NaHCO_3_, 24; NaH_2_PO_4_, 1.2; MgCl_2_, 1; glucose, 5.6; KCl, 4; CaCl_2_, 1.8; gassed with 95% O_2_/5% CO_2_ at 38 °C (pH 7.4); and supplemented with blebbistatin (10–15 μM), to suppress contraction.

### 2.2. High-Resolution Optical Mapping

**Figure 1:**
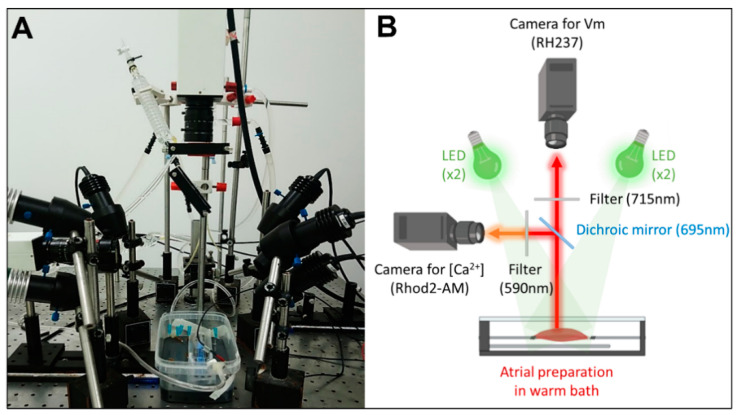
Experimental set-up for dual optical mapping of V_m_ and [Ca^2+^]_i_. (**A**) Picture and (**B**) schematic diagram of the optical design.

Preparations were imaged using dual optical mapping of the left atrial endocardial surface after being loaded with the voltage-sensitive dye RH237 (25 µM) and the Ca^2+^-sensitive dye Rhod-2 AM (0.25 µM). The endocardial surface was illuminated with monochromatic light emitting diodes (LED) at 530 nm (Mightex^®^, Pleasanton, CA, USA). Although the fluorescent probes have the same excitation band, they have different emission bands; therefore, the signals were reflected using a single 685-nm dichroic mirror (Omega, Brattleboro, VT, USA) positioned at 45°. The longest wavelength, containing the optical V_m_ signals, passed through a 715 nm long-pass filter, whereas the shortest wavelength, containing the optical Ca^2+^ signals, passed through a 590 ± 20 nm band-pass filter. The optical images (100 × 100 pixels) were acquired using 2 perpendicular Micam Ultima CMOS cameras (SciMedia USA Ltd., Costa Mesa, CA, USA) at 2 kHz with a spatial resolution of 700 µm. A schematic design of the optical setup for dual optical mapping is shown in Figure 1.

Optical signals were filtered using a low-pass frequency filter at 120 Hz, followed by spatial averaging (kernel 2.1 mm) and temporal averaging (kernel 1.5 ms).

**Figure 2:**
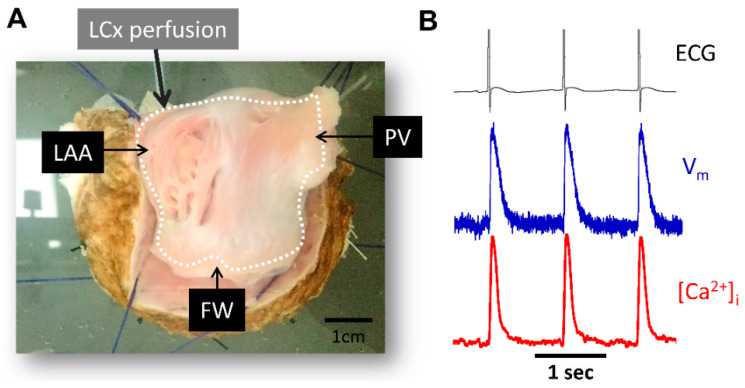
Experimental preparation and signal examples recorded during atrial pacing. (**A**) Picture of LA endocardial surface preparation. (**B**) Representative ECG signals (black), optical action potential (blue) and calcium transient signals (red) during pacing at pacing cycle length = 1000 ms. PV, pulmonary veins; LAA, left atrial appendage; FW, free-wall; LCx, left circumflex coronary artery.

Pseudo-ECG recordings across tissue preparations were recorded throughout the experiments (Figure 2). A reference electrode was positioned in the bath far from the recording electrodes.

### 2.3. Pacing Protocols

Baseline parameters were determined during LA endocardial pacing at a pacing cycle length of 1000 ms (1 Hz). Three pacing site locations were used: PV, LAA, and FW.

All of the experiments were conducted in control (Ctl) conditions (physiological saline solution) and after adding 1 µM of acetylcholine (Ach) to induce episodes of acute atrial fibrillation (pAF). If Ach did not induce AF (N = 6/15), burst pacing (10–20 Hz for 10 s) was also used (N = 9/15).

### 2.4. Chemicals

All solutions were prepared using ultrapure water supplied by a Milli-Q system (Millipore, Burlington, MA, USA). Chemicals were reagent grade and purchased from Sigma (St. Louis, MO, USA) except for Ach (Thermo Fisher Scientific, Waltham, MA, USA) blebbistatin (Tocris Bioscience, Bristol, UK) and fluorescent dye (RH237 and Rhod-2 AM; Invitrogen, Carlsbad, CA, USA).

### 2.5. Data Analysis

Data analysis was performed using two commercially available analysis software BV_Analyze (Brainvision, Tokyo, Japan); and LabChart (AD Instruments, Oxford, UK). V_m_ and Ca^2+^ datasets were spatially aligned and processed with a Gaussian spatial filter (radius 3 pixels). For the optical action potentials (APs), repolarization times at 50% and 80% return to baseline were used to calculate action potential duration (APD50 and APD80, respectively). During the acquisition, the software inverts the polarity of the Ca^2+^ transients. However, we have chosen to represent them in a conventional way, with a positive polarity in the figures of global and local data. For the optical Ca^2+^ transients, kinetics were determined by measuring the time interval between the peak of the transient and 50% of decay, defined as time to decay (TD50). The spatiotemporal dynamic between APs and Ca^2+^ transients was determined by measuring the interval between the peak of the APs and the peak of the Ca^2+^ transients (Δt to peak).

Activation maps were constructed using PV_Wave^®^ software (Perforce Software, Minneapolis, MN, USA) and Paraview (Lockheed Martin Corporation, Bethesda, MD, USA).

The pAF was analyzed by phase mapping using MATLAB^®^ (Natick, MA, USA). The cardiac electrical activity during fibrillation is a set of periodic signals; consequently, they can be characterized by their frequency and studied in terms of their distribution through the tissue and their power over a given time interval. Thus, the duration of the pAF cycles was determined by the mean dominant frequency (DF) of the tissue. This value was used to estimate the rate of local atrial activation during pAF (usually >3 Hz during fibrillation). Using DF, it was possible to calculate the regularity index (RI), which reflects the regularity between local activation waves. This parameter ensures the reliability of the DF by excluding recordings with a low RI (usually 0.2) and thus avoiding the inaccuracies associated with irregular and fractionated signals. In addition, it helped identify the areas driving the pAF [24,25]. When analyzing pAF phase maps, signals with sustained periodic activity have been classified as rotors (i.e., organized functional re-entry around a central core) if they are greater than 1 rotation (−π; +π).

### 2.6. Statistical Analysis

Data are presented as mean ± standard error of the mean (SEM) for N experiments (animals) (e.g., N Ctl; N Ach). All the statistical tests were performed using GraphPad Prism (GraphPad Software, Boston, MA, USA). First, the distribution of the data was compared to a Gaussian distribution using the Shapiro–Wilk normality test or the Kolmogorov–Smirnov test. Where the data were not normally distributed, it was transformed using log10 or reciprocal. For normally distributed data, significance was tested with the appropriate test. The effect of the site of stimulation was analyzed using a paired 1-way ANOVA followed by Tukey’s multiple comparisons tests. The effect of Ach was analyzed using a paired *t*-test followed by Tukey’s multiple comparison test. *p* < 0.05 was considered to be statistically different.

## 3. Results

### 3.1. Validation of Simultaneous Measurements of V_m_ and [Ca^2+^]_i_

**Figure 3:**
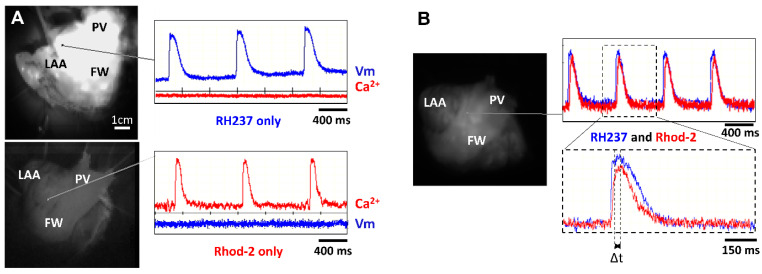
Lack of cross-talk between optical measurements of V_m_ and [Ca^2+^]_i_. (**A**) Signals recorded from two cameras to detect V_m_ and [Ca^2+^]_i_ when the LA preparation was stained with RH237 but not Rhod-2 (top panel) and Rhod-2 but not RH237 (lower panel). Note the lack of cross-talk between V_m_ and [Ca^2+^]_i_ cameras. (**B**) Signals recorded from 2 cameras to detect V_m_ and [Ca^2+^]_i_ when the LA preparation was stained with RH237 and Rhod-2 (top panel). Δt was calculated as time to peak to Ca^2+^ transient minus time to peak to AP (lower panel).

The first series of experiments were designed to verify our experimental approach for simultaneously recording optical V_m_ and [Ca^2+^]_i_ signals from the atrial endocardium. LA preparations were loaded with either RH237 or Rhod-2 AM via the left circumflex coronary artery. Figure 3 shows that LA stained with the voltage-sensitive dye RH237 exhibited null [Ca^2+^]_i_ signals in the [Ca^2+^]_i_ camera. 

Conversely, LA loaded with Rhod-2 exhibited null signals in the voltage camera array. This demonstrates a complete spectrum separation between the V_m_ and [Ca^2+^]_i_ signals.

Next, we stained LA with RH237, and Rhod-2 AM to map V_m_ and [Ca^2+^]_i_ simultaneously to check the temporal characteristics between V_m_ and [Ca^2+^]_i_. Figure 3B shows representative recordings at a PCL of 1000 ms. We observed that the AP upstroke always preceded the rise in [Ca^2+^]_i_ and the rise time of V_m_ signals was shorter than that for [Ca^2+^]_i_. The interval between the peak of the APs and the peak of the Ca^2+^ transients (Δt) can therefore be measured (Figure 3B, bottom).

### 3.2. Regional Optical Action Potential Duration and Effect of Acetylcholine

**Figure 4:**
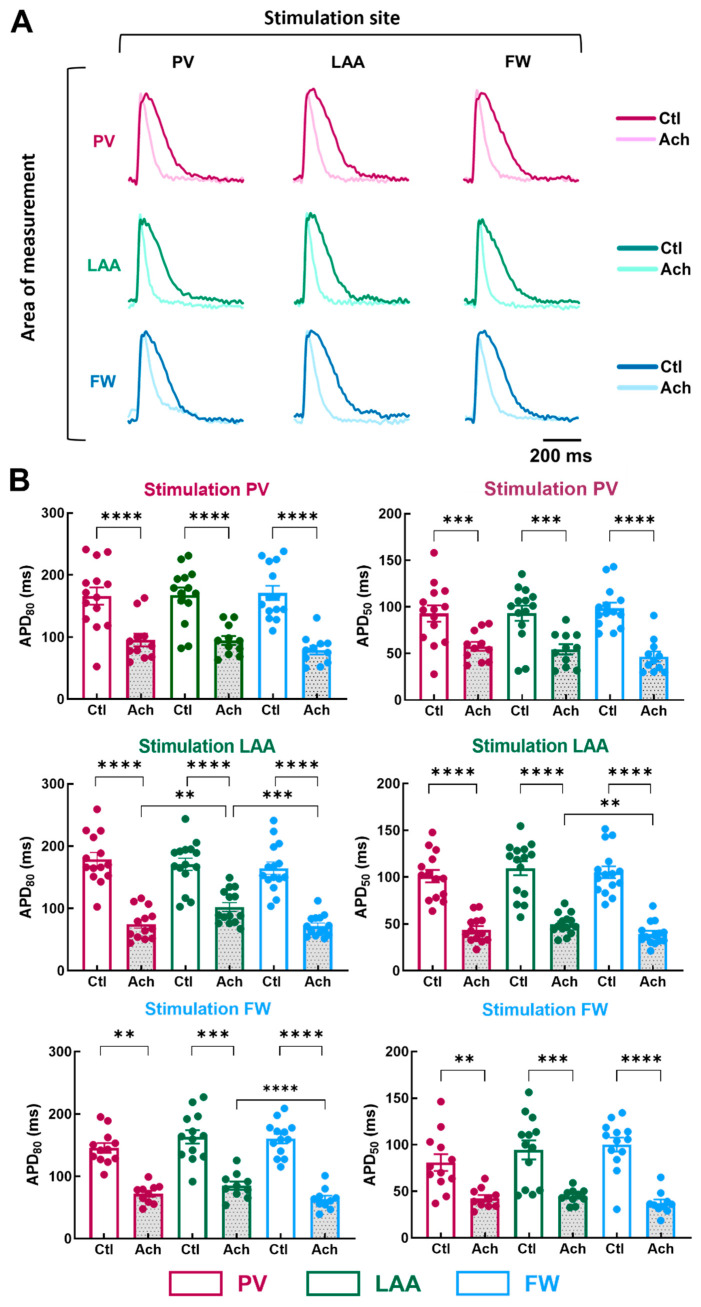
Action potential duration in three regions of the LA by pacing at three different stimulation sites. (**A**) Representative optical action potential under control perfusion (Ctl) or immediately after administration of acetylcholine (Ach 10 µM) measured in three regions (area of measurements, PV, LAA, FW, top to bottom) at three different pacing sites (PV, LAA, FW, left to right). (**B**) Mean data for action potential duration at 80% (APD_80_, left panel) and 50% (APD_50_, right panel) repolarization at three pacing sites (PV, LAA, FW, top to bottom) measured in three regions PV (purple), LAA (green), FW (blue) under control perfusion (Ctl, open bar) or after administration of acetylcholine (Ach, grey bar). N = 15; One-way ANOVA followed by a Tukey’s multiple comparison test ** *p* < 0.01, *** *p* < 0.001, **** *p* < 0.0001.

To examine the impact of the pacing site on the regional electrical properties of LA at baseline and after mimicking parasympathetic stimulation with acetylcholine (Ach), stimulation electrodes were positioned at the PV, LAA, or FW. Figure 4A illustrates representative APs recorded from myocytes stimulated at a PCL of 1000 ms.

At baseline condition, pacing site location did not cause any significant effect on APD_50_ and APD_80_ (Figure 4B; Ctl APD_80_, PV stimulation, *p* = 0.87; LAA stimulation, *p* = 0.23; FW stimulation, *p* = 0.24). In contrast, when LA preparations were perfused with 10 µM of Ach, APD differed depending on the pacing site. When LAA was stimulated, APD_80_ was longer in LAA than in PV and FW (Figure 4B; Ach, LAA: 102.3 ± 7.2 ms vs. PV: 75.1 ± 6.8 ms, *p* = 0.0085; vs. FW: 72.0 ± 4.6 ms, *p* = 0.0002), and APD_50_ was longer in LAA than in FW but not in PV (LAA: 49.8 ± 2.9 ms vs. FW: 39.4 ± 3.3 ms, *p* = 0.0014). Similarly, when the pacing site was FW, APD_80_ was significantly longer in LAA than in the FW but not in PV (Figure 4B; Ach, LAA: 85.0 ± 6.4 ms vs. FW: 63.6 ± 5.5 ms, *p* = 0.0002). Surprisingly, when the pacing site was PV, APD_80_ and APD_50_ were similar between the regions of measurements (Figure 4B). The absence of difference might involve the APD tending to increase in the PV upon PV stimulation. These results reveal subtle regional differences in action potential duration only after Ach stimulation (i.e., when action potentials are short).

### 3.3. Regional Calcium Transient Duration and Effect of Acetylcholine

**Figure 5:**
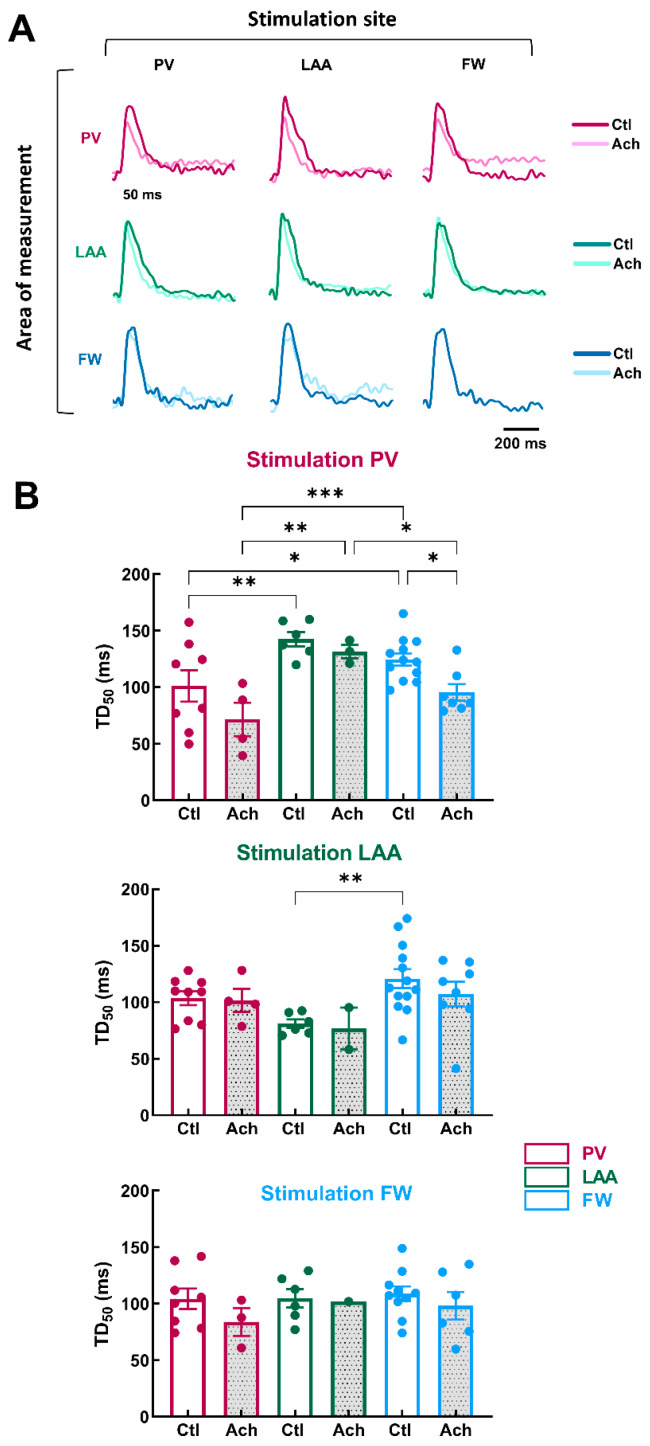
Calcium transient in three regions of LA by pacing at three different stimulation sites. (**A**) Representative optical calcium transient under control perfusion (Ctl) or immediately after administration of acetylcholine (Ach 10 µM) measured in three regions (area of measurements, PV, LAA, FW, top to bottom) at three different pacing sites (PV, LAA, FW, left to right). (**B**) Mean data for calcium transient decay time to 50% (TD_50_) at three pacing sites (PV, LAA, FW, top to bottom) measured in three regions PV (purple), LAA (green), FW (blue) under control perfusion (Ctl, open bar) or after administration of acetylcholine (Ach, grey bar). N = 13; One-way ANOVA followed by a Tukey’s multiple comparison test * *p* < 0.05, ** *p* < 0.01, *** *p* < 0.001.

Next, we investigated the impact of the site of stimulation on the regional Ca^2+^ handling properties of LA at baseline and after mimicking parasympathetic stimulation with acetylcholine (Ach, 10 µM). The electrodes of stimulation were located at PV, LAA, or FW. Figure 5A illustrates representative optical Ca^2+^ transients recorded from LA preparations stimulated at 1000 ms PCL.

In the baseline condition, when PV was stimulated, TD_50_ was significantly shorter in PV than in FW and LAA (Figure 5B, PV: = 101.1 ± 13.9 ms vs. FW: 124.6 ± 5.5 ms, *p* = 0.04 vs. LAA: 142.5 ± 6.4 ms, *p* = 0.004). When LAA was the stimulation site, TD_50_ was significantly shorter in LAA compared to FW, but there was no significant difference with PV (Figure 5B, LAA: 80.9 ± 3.8 ms vs. FW: 120.8 ± 8.5 ms, *p* = 0.003 vs. PV: 103.6 ± 6.2 ms, *p* = 0.09). When the stimulation site was FW, TD_50_ was homogeneous in the three regions (PV, LAA and FW). Taken together, these results indicate LAA showed the highest variation in terms of Ca^2+^ handling properties.

The effects of acetylcholine perfusion were modest on TD_50_. The only significant decrease was for FW when the stimulation originated from PV (Figure 5B, Ctl: 124.6 ± 5.5 ms vs. Ach: 95.4 ± 7.3 ms, *p* = 0.02). However, the difference in TD_50_ between the three regions was more pronounced during acetylcholine perfusion, especially when PV was the stimulation site.

These results indicate that Ca^2+^ homeostasis is non-homogeneous across LA. The heterogeneity of Ca^2+^ cycling means that if the trigger is localized in PV, the different regions of the LA may react discordantly, making some areas more likely to generate arrhythmias (i.e., DAD) by supporting re-entry circuits. These results are consistent with numerous studies reporting that PV is an important source of ectopic activity, triggering FA events (see Introduction).

### 3.4. Regional Differences in the Temporal Relationship between Optical V_m_ and Ca^2+^ Transient Dependent of the Site of Stimulation

**Figure 6:**
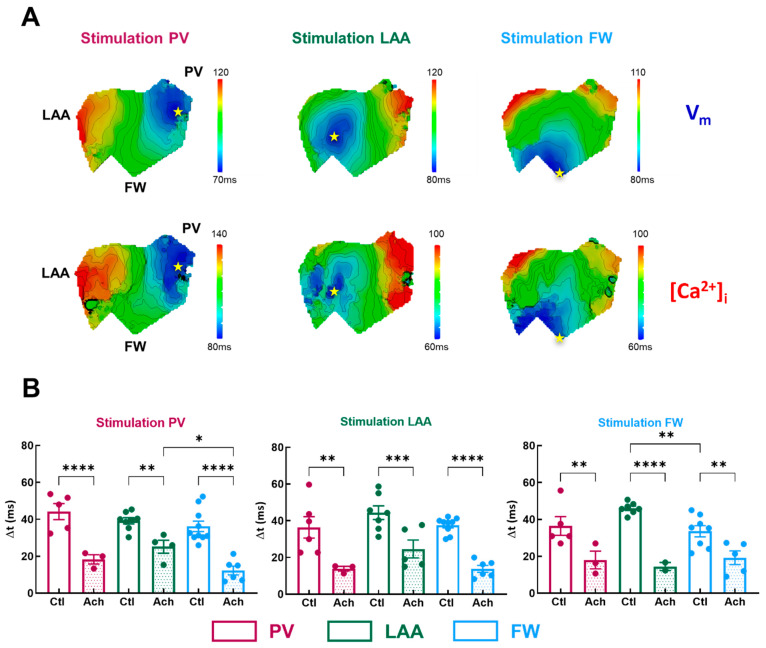
Temporal characteristics of [Ca]_i_-V_m_ relationship. (**A**) Representative activation map for V_m_ (top panel) and [Ca]_i_ (lower panel) for three pacing sites (PV, LAA, FW, left to right). The site of stimulation is represented as a yellow star. (**B**) Mean data for V_m_-Ca^2+^ delay (Δt) at three pacing sites (PV, LAA, FW, left to right) measured in three regions PV (purple), LAA (green), FW (blue) under control perfusion (Ctl, open bar) or after administration of acetylcholine (Ach, grey bar). N = 10; One-way ANOVA followed by a Tukey’s multiple comparison test * *p* < 0.05, ** *p* < 0.01, *** *p* < 0.001, **** *p* < 0.0001.

To get more insights into the potential arrhythmogenicity of these regional differences, we quantified the V_m_-Ca^2+^ delay (Δt). Simultaneous V_m_ and Ca^2+^ activation maps were compared between three regions of pacing in LA, as shown in Figure 6A. Conduction velocity appears to be slowing at the level of the transition zones between PV and LAA.

In basal condition, Δt was homogenous across all three regions for all pacing sites, except when in FW compared to the LAA (Figure 6B, FW: 33.6 ± 3.0 ms vs. LAA: 46.1 ± 1.2 ms, *p* = 0.006; vs. PV: 36.5 ± 5.1 ms *p* = 0.54) when FW was stimulated. Acetylcholine significantly shortened the V_m_-Ca^2+^ delay by −55 ± 3% on average (Figure 6B). Interestingly, when PV was stimulated, Δt was significantly shorter in FW compared to LAA but not to PV (Figure 6B, FW: 12.3 ± 2.4 ms vs. LAA: 25.2 ± 3.5 ms, *p* = 0.01, vs. PV: 18.3 ± 2.5 ms, *p* = 0.25). As a result, one can assume that if ectopic activity emanates from the PV, the FW appears to be a zone where additional rotors can emerge. Indeed, a short V_m_-Ca^2+^ delay is a measure of the arrhythmogenic susceptibility of a tissue [26]. A critical decrease in this interval indicates either the capacity of the tissue to be activated at high frequencies or an increase in the intracellular Ca^2+^ diastolic threshold, which can activate the Na^+^/Ca^2+^ exchanger in the forward mode and thus induce ectopic events.

To determine whether the difference in Δt between FW and LAA, especially upon PV stimulation, reflects either the tissue’s capacity to be activated at high frequency or a change in [Ca^2+^]_i_ homeostasis, we induced AF episodes by perfusing Ach in combination with fast stimulation trains.

### 3.5. Optical Mapping of Voltage and Calcium Dynamics during Atrial Fibrillation

Rapid pacing-induced AF episodes in LA preparation when the pacing sites were PV (5/10) and LAA (4/10). However, when FW was the pacing site, AF was induced in only one preparation (out of 10); therefore, we did not include these data in the analysis.

**Figure 7:**
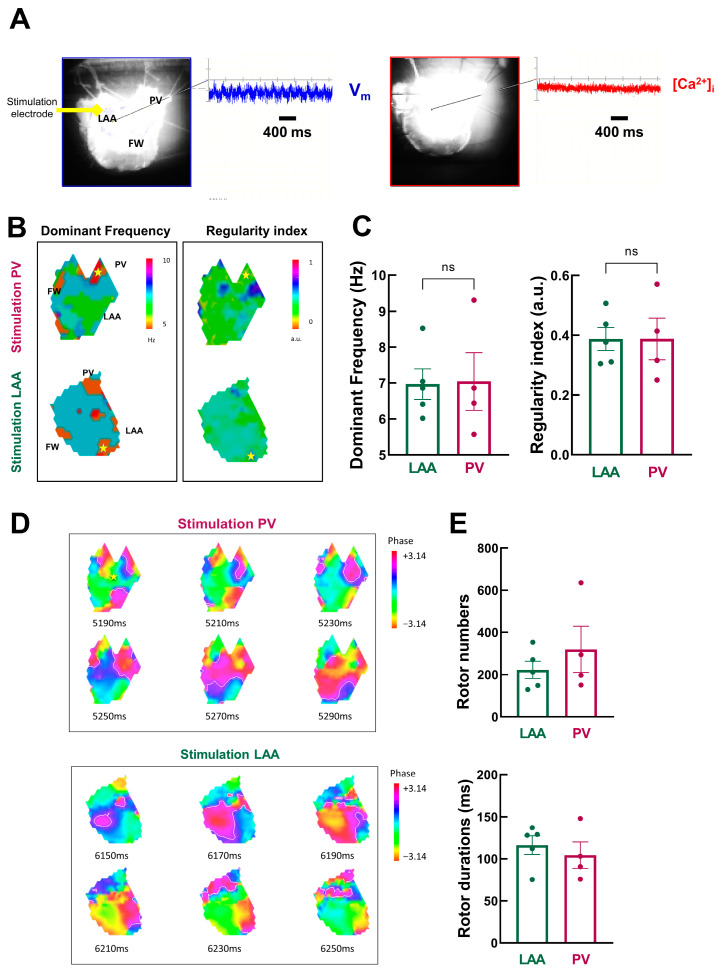
Detailed dynamic analysis of AF episodes (**A**) V_m_ and [Ca^2+^]_i_ signals during acute AF episodes. (**B**) Example of dominant frequency (left) and regularity index (right) during AF triggered at PV (top) and LAA (bottom) sites. (**C**) Mean data for dominant frequency (left) and regularity index (right) at LAA (green) and PV (purple) sites. N = 4–5; unpaired *t*-test. (**D**) Phase mapping analysis of AF episodes (snapshot of phase values with 20 ms intervals for AF episodes triggered by PV (top) and LAA (bottom) pacing). (**E**) Mean data for rotor number (top) and duration (bottom). N = 4–5; unpaired *t*-test.

Figure 7A shows an example of a typical optical recording of V_m_ and [Ca^2+^]_i_ during an AF episode.

V_m_ oscillated with a peak-to-peak amplitude that is ~47% of normal V_m_ amplitude during the pacing cycle length at 1000 ms. In contrast, [Ca^2+^]_i_ oscillations were nearly completely abolished. Analysis was therefore restricted to V_m_ signals.

We used frequency domain analysis on V_m_ signals to investigate the spatiotemporal organization of atrial fibrillation episodes. Figure 7B shows the spatial distribution of DF through LA preparation. It shows island-like regions of different dominant frequencies: the distribution of drivers is heterogeneous, with lower levels in the PV and larger levels in the LAA, notably during stimulation in PV.

However, there is no significant difference in the mean dominant frequency (Figure 7C, left, LAA: 7.0 ± 0.4 Hz vs. PV: 7.0 ± 1.6 Hz, *p* = 0.47). Likewise, the spatial distribution of the RI showed island-like regions, but on average, no significant differences between LAA and PV regions as pacing sites were observed (LAA: 0.39 ± 0.04 vs. PV: 0.39 ± 0.07, *p* = 0.50, Figure 7C right).

Next, we used phase mapping analysis to quantify rotors as AF drivers. The phase maps presented in Figure 7D show snapshots of rotors propagating through the tissue over time. Rotors are short-lived, as previously described in humans [27,28].

There is a tendency for more rotors, but with a shorter lifespan, when AF episodes are triggered from PV (Figure 7E). However, the mean data are not statistically significant (rotor number: LAA: 223 ± 41 vs. PV: 319 ± 110 *p* = 0.19; and rotor duration: LAA: 116.3 ± 11.0 ms vs. PV: 104.4 ± 15.6 ms, *p* = 0.27).

### 3.6. Stabilization of RyR2 by Dantrolene and Atrial Fibrillation Inducibility

Animal model and human atrial cardiac myocyte studies have demonstrated that diastolic Ca^2+^ leak from the sarcoplasmic reticulum can serve as a substrate for atrial arrhythmia induction and persistence (see Introduction); therefore, we next investigated the effect of dantrolene, a RyR stabilizer, on atrial fibrillation inducibility.

**Figure 8:**
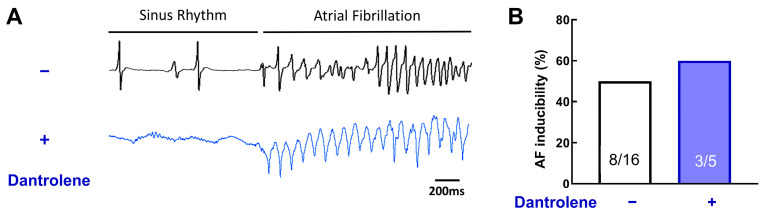
RyR stabilization does not prevent acute AF episodes. (**A**) Example of ECG traces, with or without Dantrolene, a RyR inhibitor. (**B**) Inducibility of AF for each group. Dantrolene does not change AF vulnerability. N = 16 and 5, chi-square test.

Figure 8A shows representative ECG traces of AF episodes in the absence or presence of 10 μM of dantrolene.

Surprisingly, dantrolene does not reduce AF inducibility (Figure 8B, 50% without dantrolene vs. 60% with dantrolene, *p* = 0.31). This result suggests that RyR, without post-translational *remodeling*, is not involved in AF induction.

## 4. Discussion

The purpose of this study was to determine whether Ca^2+^ signaling is homogenous throughout the different regions of the left atrium. Major findings in this study are: (1) Ca^2+^ cycling in the PV region is different from that in the LAA and FW regions, especially during short action potentials and simulation sites in this area. (2) There is no apparent relation between abnormal V_m_-Ca^2+^ delays and AF susceptibility. (3) Dantrolene, a RyR antagonist, does not prevent AF susceptibility without remodeling.

### 4.1. Experimental Approach

Simultaneous measurements of V_m_ and [Ca^2+^]_i_ have been extensively applied in single cardiac cell studies using microelectrodes or patch-clamp techniques and Ca^2+^ fluorescent indicators at the cellular level. At the tissue level, this challenging approach is less used, especially in the atria. The first data from the ventricle demonstrated that by combining V_m_-sensitive and Ca^2+^ indicator dyes with similar excitation but different emission spectra, cross-talk between V_m_ and Ca^2+^_i_ recordings could be avoided [26]. More recent studies in the rabbit ventricle have highlighted the critical nature of source-sink balance in initiating ventricular focal arrhythmias [29] and the role of ryanodine receptor refractoriness during alternans and ventricular fibrillation [30].

Careful choice of probe combinations is crucial to minimize cross-talk and maximize signal-to-noise ratios. Rhod-2 and RH-237 are classical choices and are commonly used in several species, from mice to humans [31]. Indeed, we did not see any cross-talk using this combination (Figure 3). At the atria level, to the best of our knowledge, few studies have simultaneously mapped both V_m_ and Ca^2+^_I_ in rodents, mainly mice (e.g., [32]), and only one study was performed in large mammals, sheep, like this study [21]. Rodent models do not realistically mimic the human electrophysiological phenotype in some important respects (see introduction), and the size of the atria in rodents is far from that of humans. Therefore, large-animal models are the best option for AF research since they have similar heart rates, atrial action potential morphologies, and heart sizes to humans. The sheep is the model of choice compared to pigs, where there is a high risk for ventricular fibrillation [33]. However, large animal models have important drawbacks in terms of the restricted availability of animals and higher purchase and handling costs.

### 4.2. Role of Atrial Regionalization in AF Pathophysiology

In clinics, most of the ectopic beats that initiate paroxysmal atrial fibrillation originate from the PV [6]. However, paroxysmal AF can also originate from non-PV areas (e.g., [22,34]). Recent clinical studies have identified specific electrograms in a discrete region of low-voltage areas [35,36]. At the tissue level, experimental studies have shown that re-entry and rotors occur when the effective refractory period allows atrial tissue to become re-excitable before the re-entrant impulse arrives [17]. Our results are consistent with the range of repetitive focal discharges in the initiation of AF observed in patients (cycle lengths from 110 to 270 ms) [6]. Animal studies have provided important insight into the initiation, maintenance, and progression of AF [33]. In addition, there have been extensive studies of atrial cardiomyocytes from AF patients [9]. At the cellular level, the re-entry-promoting shortening of APD seen with pAF is due to the reduced L-type Ca^2+^ current and increased repolarizing currents such as *I*_K1_ and *I*_K,ACh_ [37]. The increase in inward-rectifier K^+^ currents hyperpolarizes the resting membrane potential, which enhances excitability and stabilizes spiral-wave re-entry [38]. Our data with ach infusion are consistent with these observations.

### 4.3. Calcium Regional Heterogeneity and Vulnerability to Focal Arrhythmia

Triggered cardiac activity results from early- or delayed after-depolarizations (EADs and DADs, respectively, see introduction). These ectopic foci can trigger AF-maintaining re-entry in a vulnerable substrate or, if they fire repeatedly, act as AF-maintaining drivers. These Ca^2+^ cycling alterations at the cellular level can trigger ectopic activity at the tissue level if a sufficient number of adjacent cells are altered at the same time [39]. Increased sarcoplasmic reticulum Ca^2+^ leaks resulting from RyR2 dysfunction or sarcoplasmic reticulum Ca^2+^ overload can activate NCX, resulting in a transient-inward current that promotes EADs and DADs [12]. Our study shows that Ca^2+^ cycling differences across the healthy LA may contribute to a unique, region-dependent Ca^2+^ signaling and thus be involved in the pro-arrhythmic activity associated with the electrical dysfunctions observed during AF. Our results support the hypothesis that when Ca^2+^ cycling is dysregulated, ectopic activity occurs.

A short V_m_-Ca^2+^ activation delay is indicative of Ca^2+^-mediated focal activity. During normal excitation-contraction coupling, atrial V_m_-Ca^2+^ delay was ≈20 ms when Ach was infused, within the range of a previous report on atria in a sheep model myocardial infarction of the LA [21]. This delay is longer (≈8 ms) than that reported in ventricular preparation in rabbits [26,29] and humans [40]. Disappointingly, our findings do not provide insights into the interpretation of intracellular Ca^2+^ signals during AF. Our dual optical mapping data of V_m_ and [Ca^2+^]_i_ show peak-to-peak oscillation during AF that is ~20% of the amplitude of the normal intracellular Ca^2+^ transient. This is less than what is observed in ventricular fibrillation using a similar approach (dual optical mapping, ~30%) [30,41]. It may be related to the size of the Ca^2+^ transient, which is smaller in the atria than in the ventricles [42] and the Ca^2+^ silencing phenomenon observed at the cellular level [43]. However, in our study, the colocalization on voltage and Ca^2+^ maps in the LA suggests a causal link between the regional Ca^2+^ cycling, DADs, and triggered ectopic activity.

In our study, we showed that dantrolene does not prevent AF episodes in sheep. However, dantrolene did appear to suppress AF induction in a sheep model of left myocardial infarction [21]. This observation is compatible with the notion that long-term remodeling is necessary to initiate AF-maintaining reentry with Ca^2+^ mishandling as a trigger. In addition, acute episodes of AF, in the absence of stress (e.g., ischemia or hypertension), might not involve a dysregulation of the RyR. Further work is needed to evaluate Ca^2+^ cycling at the cellular level, e.g., calcium sparks, including in the presence of dantrolene, but also using techniques such as western blotting and immunocytochemistry.

### 4.4. Limitations

One limitation of the current study is that AF was induced acutely in sheep hearts by Ach infusion and rapid pacing, which may not be relevant to persistent or chronic forms of AF in humans. Indeed, the acute model of AF does not incorporate pathophysiological structural remodeling, which could conceivably compound the degree of desynchrony observed across the transmural wall. However, in the clinical electrophysiology laboratory, atrial pacing is often performed to induce paroxysmal AF in patients. Because more than 70% of pAF is thought to be driven by enhanced spontaneous activity originating in the PV, rapid atrial pacing may be most relevant to uncovering increased susceptibility to pAF in humans [44].

## 5. Conclusions

In this study, we evaluated the tissular determinants of intracellular Ca^2+^ handling in different regions of the LA of the sheep, an animal relevant to human pathophysiology. The underlying heterogeneities appeared to enhance Ca^2+^ cycling, with relevance to atrial fibrillation susceptibility. The novel experimental insights we obtained into fundamental arrhythmogenic mechanisms in AF may facilitate the development of safer and more effective mechanism-based therapeutic strategies.

## Data Availability

The data supporting the study findings can, upon reasonable request, be made available from the corresponding author.
